# Comorbid Chronic Diseases are Strongly Correlated with Disease Severity among COVID-19 Patients: A Systematic Review and Meta-Analysis

**DOI:** 10.14336/AD.2020.0502

**Published:** 2020-05-02

**Authors:** Hong Liu, Shiyan Chen, Min Liu, Hao Nie, Hongyun Lu

**Affiliations:** ^1^Department of Nutrition, the Third Xiangya Hospital of Central South University, Changsha, China.; ^2^Department of Endocrinology & Metabolism, the Fifth Affiliated Hospital of Sun Yat-sen University, Zhuhai, China.; ^3^Department of Geriatrics, the First Affiliated Hospital of Hunan Normal University, Changsha, China.; ^4^Department of Endocrinology & Metabolism, Zhuhai Hospital Affiliated with Jinan University, Zhuhai People’s Hospital, Zhuhai, China.

**Keywords:** coronavirus disease 2019 (COVID-19), diabetes, cardiovascular diseases, hypertension, chronic pulmonary disease, meta-analysis

## Abstract

Coronavirus disease 2019 (COVID-19) has resulted in considerable morbidity and mortality worldwide since December 2019. In order to explore the effects of comorbid chronic diseases on clinical outcomes of COVID-19, a search was conducted in PubMed, Ovid MEDLINE, EMBASE, CDC, and NIH databases to April 25, 2020. A total of 24 peer-reviewed articles, including 10948 COVID-19 cases were selected. We found diabetes was present in 10.0%, coronary artery disease/cardiovascular disease (CAD/CVD) was in 8.0%, and hypertension was in 20.0%, which were much higher than that of chronic pulmonary disease (3.0%). Specifically, preexisting chronic conditions are strongly correlated with disease severity [Odds ratio (OR) 3.50, 95% CI 1.78 to 6.90], and being admitted to intensive care unit (ICU) (OR 3.36, 95% CI 1.67 to 6.76); in addition, compared to COVID-19 patients with no preexisting chronic diseases, COVID-19 patients who present with either diabetes, hypertension, CAD/CVD, or chronic pulmonary disease have a higher risk of developing severe disease, with an OR of 2.61 (95% CI 1.93 to 3.52), 2.84 (95% CI 2.22 to 3.63), 4.18 (95% CI 2.87 to 6.09) and 3.83 (95% CI 2.15 to 6.80), respectively. Surprisingly, we found no correlation between chronic conditions and increased risk of mortality (OR 2.09, 95% CI 0.26 to16.67). Taken together, cardio-metabolic diseases, such as diabetes, hypertension and CAD/CVD were more common than chronic pulmonary disease in COVID-19 patients, however, each comorbid disease was correlated with increased disease severity. After active treatment, increased risk of mortality in patients with preexisting chronic diseases may reduce.

The recent outbreak of COVID-19 has been rapidly spreading on a global scale[1]. On January 30, 2020, the World Health Organization (WHO) declared that the epidemic of COVID-19 was a public health emergency of international concern (PHEIC). COVID-19 has resulted in considerable morbidity and mortality in more than 200 countries and regions worldwide. At present, the number of patients with COVID-19 is rapidly rising to nearly 3 million, and its harm to human beings has exceeded the outbreak of severe acute respiratory syndrome (SARS) in 2002 and Middle East respiratory syndrome (MERS) in 2012 [2]. As of April 29, 2020, more than 200,000 people all over the world lost their life because of the infection of SARS-CoV-2, with a crude death rate of 6.95%.

COVID-19 is general susceptibility [3-7] and is accompanied by a cluster of flu-like symptoms [6] and life-threatening severe illnesses including acute respiratory distress syndrome (ARDS), acute kidney injury, myocarditis, and organ failure [6, 8]. It is believed that middle-aged and elderly patients with chronic diseases, such as diabetes, cardiovascular diseases and hypertension are susceptible to respiratory failure and may have a poorer outcome [6, 8]. Evaluating the prevalence of these chronic conditions is fundamental to mitigate COVID-19 complications and mortality. However, this effort has been hindered by the limited number of cases in some studies, and the varying study designs [9-11].

This present study was aimed to provide a systematic evaluation and detailed estimate on the prevalence and effects of preexisting chronic conditions in COVID-19 patients. This assessment may aid the public health sector while developing policies for surveillance, preparedness, and response to COVID-19 and its severe outcomes.

## MATERIALS AND METHODS

### Search strategy and selection criteria

A search was conducted in PubMed, Ovid MEDLINE, EMBASE, CDC, and NIH databases to 25 April, 2020 using the search terms (MeSH) ‘‘Coronavirus disease 2019’’, ‘‘2019 novel coronavirus’’, ‘‘COVID-19’’, ‘‘2019-nCov’’, or ‘‘SARS Cov-2’’ AND ‘‘Diabetes”, “Hypertension”, “Chronic pulmonary disease” or “Cardiovascular disease”. The search was limited to articles describing the epidemiological, demographic, clinical features, outcomes and reporting the prevalence of chronic diseases in COVID-19 patients. Reports published as review articles, letters, case studies, editorials, conference abstracts, vaccination trials, family-based studies, and articles without abstracts were excluded.

**Figure 1.**
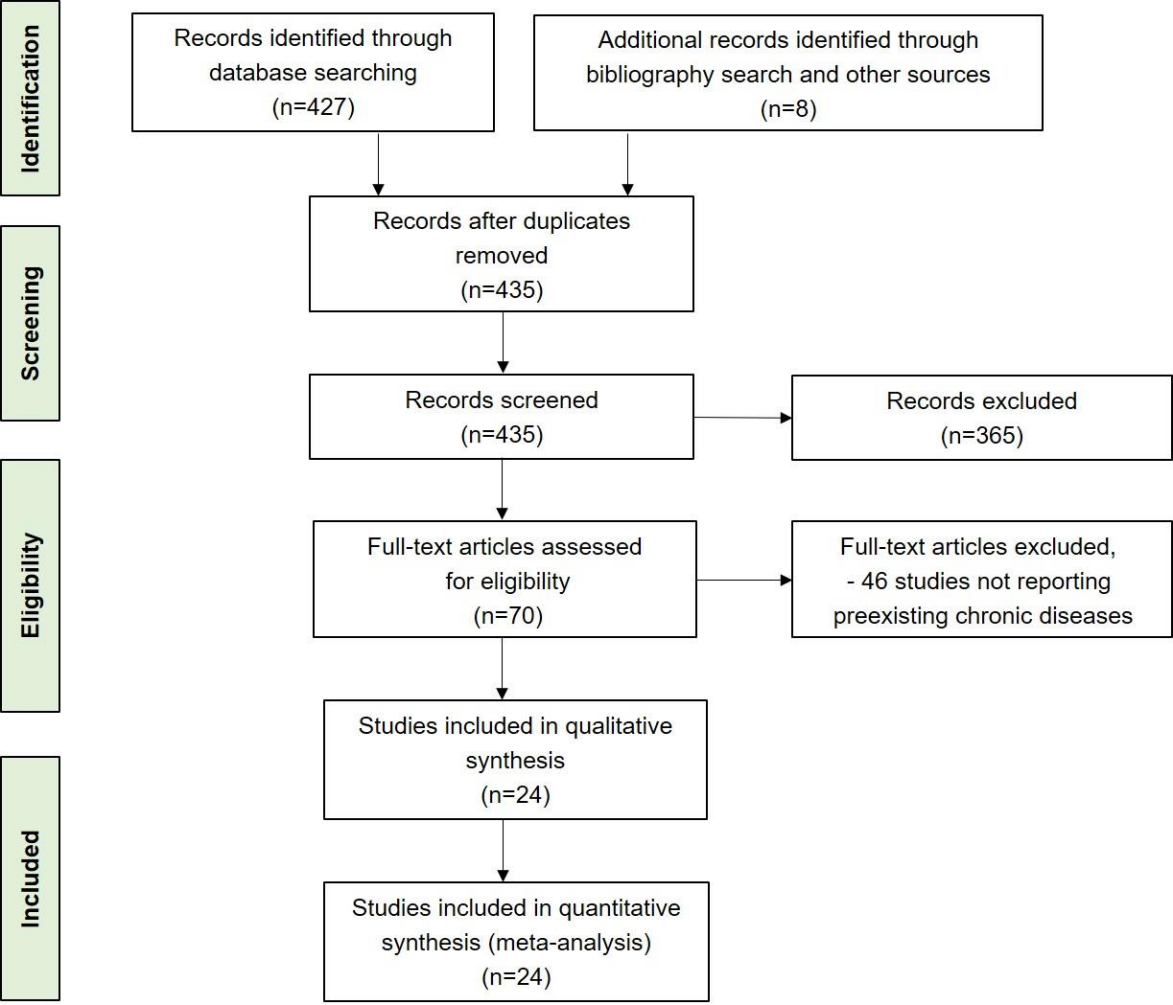
**Systematic literature review process.** The flow diagram describes the systematic review of the literature for the proportion of comorbid chronic diseases in patients with COVID-19.

The search identified 427 records; an additional eight reports were identified from a search of the bibliographies of previously obtained articles and other sources such as Google, Google Scholar, and the AMED (Allied and Complementary Medicine) search engine, for a total of 435 records. Following screening for duplicate records, the 435 records were all retained for abstract scanning against the above-mentioned inclusion/exclusion criteria. The abstracts of the identified studies were reviewed independently by both authors. Differences were resolved through discussion, until a consensus was reached. The abstract review resulted in the exclusion of 365 records. Full-text retrieval and review were conducted for the remaining 70 articles. 46 studies were then eliminated based upon the above selection criteria (Fig. 1). The percentage agreement on the inclusion between the two reviewers was 90%, with Cohen’s kappa statistic k = 0.80 [95% confidence interval (95% CI) 0.75 to 0.89]. A total of 24 peer-reviewed articles were selected for the present study (Table 1) [3-6, 8, 12-30].

**Table 1:** Characteristics of the included studies and meta-analysis of the clinical symptoms and comorbid chronic diseases in patients with COVID-19.

Study[ref]^a^	NOS	Dates (mm. yy)	n			Age (years)	Age ≥50 years (%)	Symptoms (%)					Comorbidities(%)			
			All	M	F			fever	cough	fatigue or myalgia	shortness of breath or dyspnea	diarrhea	diabetes	hypertension	CAD/CVD	chronic pulmonary disease
Guan et al.,2020 ^[3]^	8	12.19-01.20	1099	640	459	47.0	44.0	88.7	67.8	38.1	18.7	3.8	7.4	15.0	2.5	1.1
Chen et al.,2020 ^[4]^	7	01.20-01.20	99	67	32	55.5	67.7	82.8	81.8	11.0	31.3	2.0	13.0	^b^	40.0	1
Huang et al.,2020 ^[18]^	6	12.19-01.20	41	30	11	49.0	48.8	98.0	76.0	44.0	55.0	3.0	20.0	15.0	15.0	2.0
Liu et al.,2020 ^[8]^	7	12.19-01.20	137	61	76	55.0	^b^	81.8	48.2	32.1	19.0	8.0	10.2	9.5	7.3	1.5
Shi et al.,2020 ^[22]^	6	12.19-01.20	81	42	39	49.5	49.4	73.0	59.0	^b^	42.0	4.0	12.0	15.0	10.0	11
Song et al.,2020 ^[20]^	6	01.20-01.20	51	25	26	49.0	47.1	96.0	47.0	31.0	14.0	10.0	6.0	10.0	2.0	2.0
Yang et al.,2020 ^[6]^	7	12.19-01.20	52	35	17	59.7	55.0	98.0	77.0	11.5	63.5	^b^	17.0	^b^	10.0	8
Xu et al.,2020 ^[19]^	7	01.20-01.20	62	35	27	41.0	^b^	77.0	81.0	52.0	^b^	8.0	2.0	8.0	^b^	2.0
Zhang et al.,2020 ^[15]^	8	01.20-02.20	140	71	69	57.0	70.0	78.6	64.3	64.3	31.4	12.9	12.1	30.0	5.0	2.8
Wu et al.,2020 ^[16]^	7	01.20-02.20	80	39	41	46.0	35.0	78.8	63.8	22.5	37.5	1.3	6.3	^b^	31.3	1.25
Hu et al.,2020 ^[14]^	6	01.20-02.20	24	8	16	32.5	37.5	20.8	8.3	8.3	^b^	^b^	8.3	8.3	4.2	0.0
Huang et al.,2020 ^[17]^	7	12.19-01.20	34	14	20	56.2	^b^	94.1	50.0	64.7	14.7	14.7	11.8	23.5	17.6	8.8
Yang et al.,2020 ^[13]^	8	01.20-02.20	149	81	68	45.1	^b^	76.5	58.4	3.4	1.3	7.4	6.0	^b^	18.8	0.7
Wang et al.,2020 ^[5]^	7	01.20-01.20	138	75	63	56.0	^b^	98.6	59.4	69.6	31.2	10.1	10.1	31.2	14.5	2.9
Xu et al.,2020 ^[21]^	7	01.20-02.20	90	39	51	50.0	^b^	78.0	63.0	28.0	^b^	6.0	6.0	19.0	3.0	1.0
Li et al.,2020 ^[12]^	7	01.20-02.20	83	44	39	45.5	^b^	86.7	78.3	18.1	10.8	8.4	7.8	6.0	1.2	6.0
Shi et al.,2020 ^[23]^	8	01.20-02.10	416	205	211	64	^b^	80.3	34.6	13.2	28.1	3.8	14.4	30.5	10.6	2.9
Bhatraju et al.,2020^[24]^	6	02.20-03.20	24	15	9	64	^b^	50.0	88.0	^b^	88.0	^b^	58.0	^b^	^b^	16.7
Feng et al.,2020 ^[25]^	7	01.20-02.20	476	271	205	53	^b^	81.9	56.5	11.6	22.9	10.3	10.3	23.7	8.0	4.6
Du et al.,2020 ^[26]^	8	12.19-02.20	179	97	82	57.6	72.6	98.9	81.6	39.7	49.7	21.8	18.4	32.4	16.2	4.5
Liu et al.,2020 ^[27]^	6	12.19-01.20	78	39	39	38	^b^	73.1	43.6	^b^	^b^	^b^	6.4	10.3	^b^	2.6
Grasselli et al.,2020 ^[28]^	8	02.20-03.20	1591	1304	287	63	87.2	^b^	^b^	^b^	^b^	^b^	11.3	32.0	14.0	2.6
Richardson et al.,2020^[29]^	8	03.20-04.20	5700	3437	2263	63	78.5	30.4	^b^	^b^	17.3	^b^	31.8	53.0	16.9	8.4
Simonnet et al.,2020^[30]^	7	02.20-04.20	124	90	34	60	1.00	^b^	^b^	^b^	^b^	^b^	22.6	48.4	^b^	^b^
Total/Overall	12.19-04.20	10948	6764	4184	52.4^c^ (32.5-64.0)^d^											
Prevalence^e^							59.0	79.0	61.0	32.0	31.0	7.0	10.0	20.0	8.0	3.0
95% CI							49.0-68.0	65.0-92.0	54.0-69.0	21.0-43.0	25.0- 37.0	5.0-9.0	8.0-12.0	15.0-26.0	3.0-12.0	1.0-3.0
*I^2^* (%)							98.5	99.7	95.1	98.5	97.4	81.1	73.0	94.2	98.0	94.5

mm, month; yy, year; M, male; F, female; SE, standard error; CI, confidence interval, NOS, Newcastle-Ottawa Scale;

a ^[3]- [8], [12]-[23], [25]-[27] ^Studies were from China; ^[24], [29] ^studies were from America; ^[28] ^study was from Italy; ^[30] ^study was from France.

b Any empty cells represents the absence of data in the original text.

c the median age [IQR].

d Age range.

e Meta-analysis for the prevalence was calculated from binary random-effects model analysis.

f * p *< 0.001.

### Data Extraction and Quality Assessment

We used Newcaltle-Ottawa scale (NOS) (Table 1) for evaluating the quality of the included studies and Preferred Reporting Items for Systematic Reviews and Meta-Analyses (PRISMA) standards (Supplementary Table 1) for abstracting data and assessing data quality and validity. Independent extraction was conducted by two observers. NOS scores of at lease six were considered high-quality literature.

The prevalence of preexisting chronic diseases including diabetes, hypertension, coronary artery disease/cardiovascular disease (CAD/CVD) and chronic pulmonary disease, together with clinical symptoms such as fever, cough, fatigue or myalgia and shortness of breath or dyspnea were extracted from the identified studies. The primary outcome measure was the prevalence of comorbid chronic diseases in COVID-19 patients and the correlation between comorbid chronic diseases and adverse outcomes [increased risk of disease severity, admittance to intensive care unit (ICU), and mortality].

### Statistical Analysis

All analyses were performed using Stata/SE 16.0. Results about the correlation between comorbid chronic diseases and adverse outcomes were expressed as pooled odds ratios (OR), and 95% CI. OR values > 1 represent a direct association and < 1 an inverse association. The size of the squares was correlated with the weight of the selected study [31].

The results of the included studies were performed with fixed/random-effect models. We used the *I*^2^ statistics to assess the magnitude of heterogeneity: 25%, 50%, and 75% represented low, moderate, and high degrees of heterogeneity, respectively. The chosen of the proper effect model was based on the analysis results: the fixed effect model was used if *I*^2^ < 50% and the random effect model was used if *I*^2^ ≥ 50% [31].

The potential for publication bias was addressed by drawing Begg funnel plots and Egger’s linear regression test plots; and *p*<0.05 indicates obvious publication bias.

## RESULTS

### Characteristics of the included studies and meta-analysis of the clinical symptoms in patients with COVID-19

A total of 24 studies were included in our final analysis [3-6, 8, 12-30], with a total of 10948 COVID-19 patients(Table 1). Among them, 20 studies were from China, 2 from United States, 1 from Italy, and 1 from France. The number of cases in the selected studies varied by approximately 238-fold and ranged from 24 [14, 24] to 5700 [29] cases. The sex ratio (male to female) was 1.62 and the overall median age of the subjects was 52.4 years (range 32.5 to 64.0 years) (Table 1). Meta-analysis also suggests that people aged more than 50 years occurred 59.0 % (95% CI 49.0% to 68.0 %) in patients with COVID-19 (Table 1).

Meta-analysis of the included studies showed that the most prevalent clinical symptoms were fever (79.0 %, 95% CI 65.0% to 92.0%) and cough (61.0%, 95% CI 54.0% to 69.0%), followed by fatigue or myalgia (32.0%, 95% CI 21.0% to 43.0%), shortness of breath or dyspnea (31.0%, 95% CI 25.0% to 37.0%) and diarrhea (7.0%, 95% CI 5.0% to 9.0%). There was significant heterogeneity (Cochran’s Q) in the estimates of clinical symptoms among the examined studies (*p*<0.05) with an *I*^2^ index varying from 81.1% to 99.7% (Table 1).

### Characteristics of the included studies grouped by disease severity in patients with COVID-19

Among the 10948 cases, 2699 cases were severe patients (Table 2). The sex ratio (male to female) in severe and non-severe group has no statistical significance (the ratio is 2.66 and 1.21, respectively, *p*=0.55), and the median age of the subjects in severe group was older than non-severe group [60.9 years (range 45.0 to 74.0 years) vs 43.8 years (range 37.0 to 60.0 years), *p*=0.003] (Table 2).

Meta-analysis of the identified studies showed that the incidence of fever, cough and fatigue or myalgia in severe patients was higher than that in mild patients, but there was no significant difference (*p* was 0.28, 0.10 and 0.24, respectively). The incidence of shortness of breath or dyspnea in severe patients was significantly higher than that in mild patients (*p*=0.006). The incidence of diarrhea was the same in the two groups (*p*=0.18) (Table 2).

**Table 2:** Characteristics of the included studies grouped by severe and non-severe cases and meta-analysis of the clinical symptoms and comorbid chronic diseases in patients with COVID-19.

Study[ref]^a^	n								Symptoms(%)										Comorbidities(%)							

	non-severe				severe				fever		cough		fatigue or myalgia		shortness of breath or dyspnea		diarrhea		diabetes		hypertension		CAD/CVD		chronic pulmonary disease	

	All	M	F	age	All	M	F	age	mild	severe	mild	severe	mild	severe	mild	severe	mild	severe	mild	severe	mild	severe	mild	severe	mild	severe
Guan et al.,2020 ^[3]^	926	540	386	45.0	173	100	73	52.0	88.1	91.9	67.3	70.5	37.8	39.9	15.1	37.6	3.5	5.8	5.7	16.2	13.5	23.7	1.8	5.8	0.6	3.5
Huang et al.,2020^[18]^	28	19	9	49.0	13	11	2	49.0	96.0	100.0	71.0	85.0	39.0	54.0	37.0	92.0	4.0	0.0	25.0	8.0	14.0	15.0	11.0	23.0	0.0	8.0
Yang et al.,2020 ^[6]^	20	14	6	51.9	32	21	11	64.6	100.0	97.0	75.0	78.0	10.0	12.5	60.0	66.0	^b^	^b^	10.0	22.0	^b^	^b^	10.0	9.0	10.0	6.0
Xu et.al.,2020 ^[19]^	29	16	13	39	33	19	14	45	83	73	79	82	45	58	^b^	^b^	0	9	0.0	3.0	3.0	12.0	^b^	^b^	0	3.0
Zhang et al.,2020^[15]^	82	38	44	51.5	58	33	25	64.0	72.0	87.9	54.9	77.6	62.2	67.2	24.4	41.4	11.0	15.5	11.0	13.8	24.4	37.9	3.7	6.9	0.0	3.4
Wang et al.,2020 ^[5]^	102	53	51	51.0	36	22	14	66.0	98.0	100.0	59.8	58.3	35.3	33.3	19.6	63.9	7.8	16.7	5.9	22.2	21.6	58.3	10.8	25.0	1.0	8.3
Li et al.,2020^[12]^	58	29	29	41.9	25	15	10	53.7	86.2	88.0	70.7	96.0	17.2	20.0	3.4	28.0	8.6	8.0	0.0	28.0	5.2	8.0	0.0	4.0	1.7	16.0
Shi et al.,2020 ^[23]^	334	161	173	60	82	44	38	74	81.1	76.8	34.7	34.1	12	18.3	27.2	31.7	4.5	1.2	12	24.4	23.4	59.8	6	29.3	1.8	8.5
Bhatraju etal.,2020^[24]^	^b^	^b^	^b^	^b^	24	15	9	64.0	^b^	50.0	^b^	88.0	^b^	^b^	^b^	88.0	^b^	^b^	^b^	58.0	^b^	^b^	^b^	^b^	^b^	16.7
Feng et al.,2020 ^[25]^	^b^	^b^	^b^	^b^	476	271	205	^b^	0	85.9	0	59.4	0	12.6	0	24.4	0	11.0	0	10.3	0	23.7	0	8.0	0	4.6
Du et al.,2020 ^[26]^	158	87	71	56	21	10	11	70.2	98.7	100	83.5	66.7	36.7	61.9	44.9	85.7	19.6	38.1	17.1	28.6	28.5	61.9	10.8	57.1	5.1	0
Liu et al.,2020 ^[27]^	67	32	35	37	11	7	4	66	^b^	^b^	44.8	46.4	^b^	^b^	^b^	^b^	^b^	^b^	4.5	18.2	9	18.2	^b^	^b^	1.5	9.1
Grasselli et al.,2020^[28]^	^b^	^b^	^b^	^b^	1591	1304	287	63	^b^	^b^	^b^	^b^	^b^	^b^	^b^	^b^	^b^	^b^	^b^	11.3	^b^	32	^b^	14	^b^	2.6
Simonnet et al.,2020^[30]^	^b^	^b^	^b^	^b^	124	90	34	60	^b^	^b^	^b^	^b^	^b^	^b^	^b^	^b^	^b^	^b^	^b^	22.6	^b^	48.4	^b^	^b^	^b^	^b^
Total/Overall	1840	989	817	43.8^c^ (37.0-60.0)^d^	2699	1962	737	60.9^c^ (45.0-74.0) ^d^																		
Prevalence^e^									90.0	99.0	64.0	71.0	32.0	37.0	27.0	55.0	8.0	8.0								
95% CI									86.0-95.0	98.0-100.0	52.0-75.0	60.0-81.0	21.0-44.0	24.0-50.0	17.0-36.0	39.0-71.0	4.0-11.0	4.0-13.0								
Q^f^																										
*I^2^* (%)									95.5	94.0	95.4	91.7	95.6	94.2	94.4	95.9	81.2	92.5								

M, male; F, female; SE, standard error; CI, confidence interval.

a ^[3], [18], [6], [15], [5], [12], [19], [23], [25], [26], [27] ^Studies were from China; ^[24] ^study was from America; ^[28] ^study was from Italy; ^[30] ^study was from France.

b Any empty cells represents the absence of data in the original text.

c the median age [IQR].

d Age range.

e Meta-analysis for the prevalence was calculated from binary random-effects model analysis.

f * p *< 0.001.

**Figure 2.**
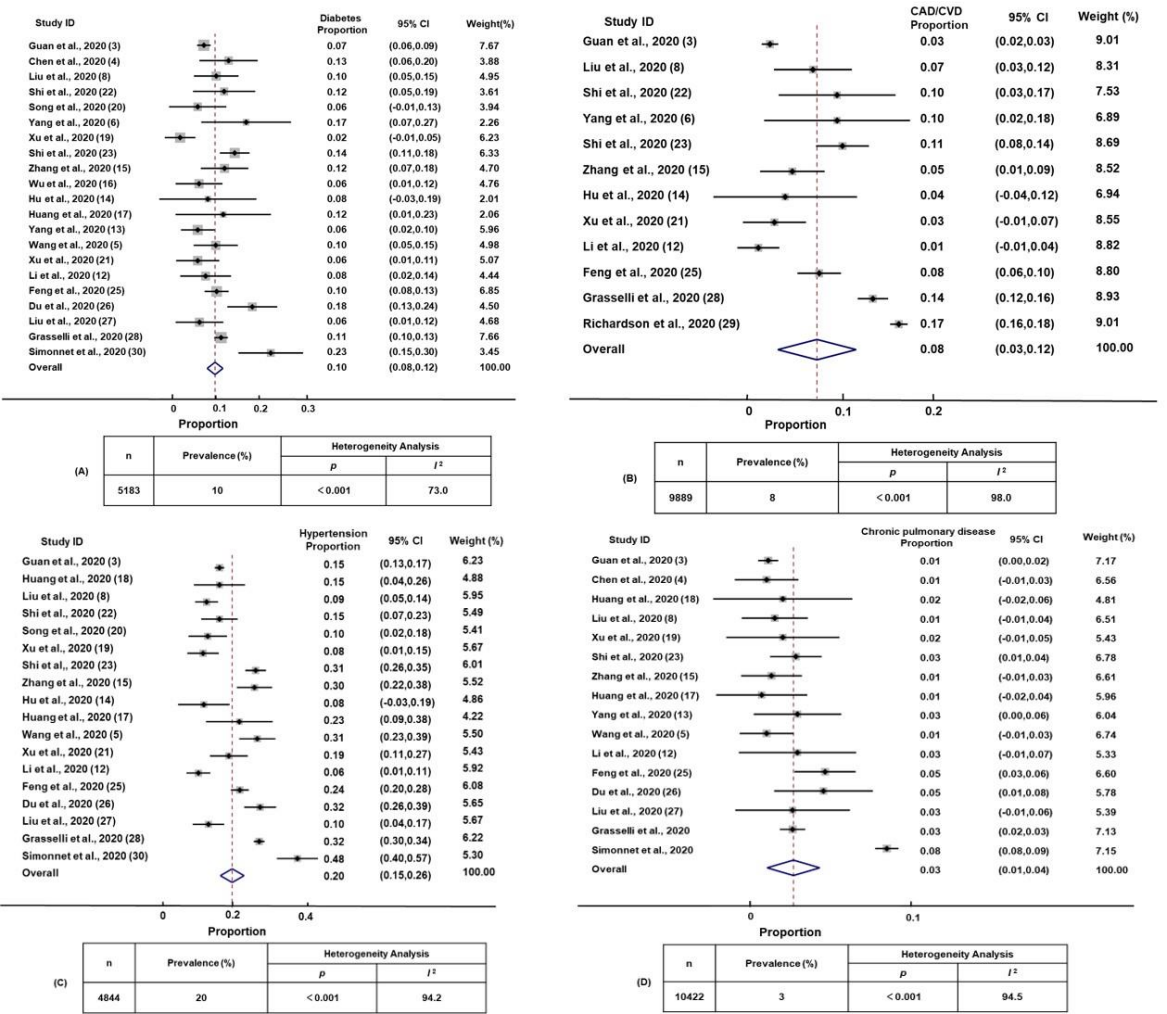
**The proportions of comorbid chronic diseases in patients with COVID-19.** Forest plot showing the proportion of comorbid diabetes (**A**), coronary artery disease/cardiovascular disease (CAD/CVD) (**B**), hypertension (**C**), and chronic pulmonary disease (**D**) in SARS-CoV-2-infected patients. Weights were calculated from random-effects model analyses. The size of the squares reflects the relative weight of each study in the meta-analysis. Inserts within each panel show the total number of subjects analyzed (n) and prevalence (%) of the comorbid diseases (%), together with heterogeneity analysis carried out using the Q test and the among-studies variation (*I^2^* index).

### The incidence of comorbid chronic diseases in patients with COVID-19

As shown in Fig. 2 (inserts A and B), diabetes was present in 10.0% (95% CI 8.0% to 12.0%) and CAD/CVD was prevalent in 8.0% (95% CI 3.0% to 12.0%) of the patients. Hypertension was present in 20.0% (95% CI 15.0% to 26.0%) and chronic pulmonary disease in 3.0% (95% CI 1.0% to 4.0%) (Fig. 2, inserts C and D). The proportions of diabetes, hypertension, CAD/CVD and chronic pulmonary disease varied by 11.5-, 8.0-, 17.0- and 8.0-fold, respectively, among the identified studies. This wide among-studies variation in the proportion of comorbid diseases may have resulted in the significant heterogeneity (Cochran’s Q) observed for estimates of diabetes, hypertension, CAD/CVD and chronic pulmonary disease (*p *< 0.001), with an *I*^2^ index ranging from 73.0% to 98.0% (Fig. 2).

### The effects of comorbid chronic diseases on the clinical outcomes in patients with COVID-19

We screened out articles that included the numbers of death, disease severity, or admittance to ICU, which allowed us to pool their data into a further analysis. Next, we performed a meta-analysis in order to examine the association between preexisting chronic diseases and clinical outcomes (disease severity, admittance to ICU and mortality rate) in patients with COVID-19. We found that preexisting chronic diseases were strongly correlated with increased disease severity (OR 3.50, 95% CI 1.78 to 6.90), with moderate heterogeneity (*I*^2^=60.9%); increased admittance to ICU (OR 3.36, 95% CI 1.67 to 6.76), with low heterogeneity (*I*^2^=36.0%) (Supplementary Fig. 1, inserts A and B). To our surprise, our analysis revealed that preexisting chronic diseases were not significantly correlated with COVID-19 mortality (OR 2.09, 95% CI 0.26 to 16.67) (Supplementary Fig. 1, inserts C).

**Figure 3.**
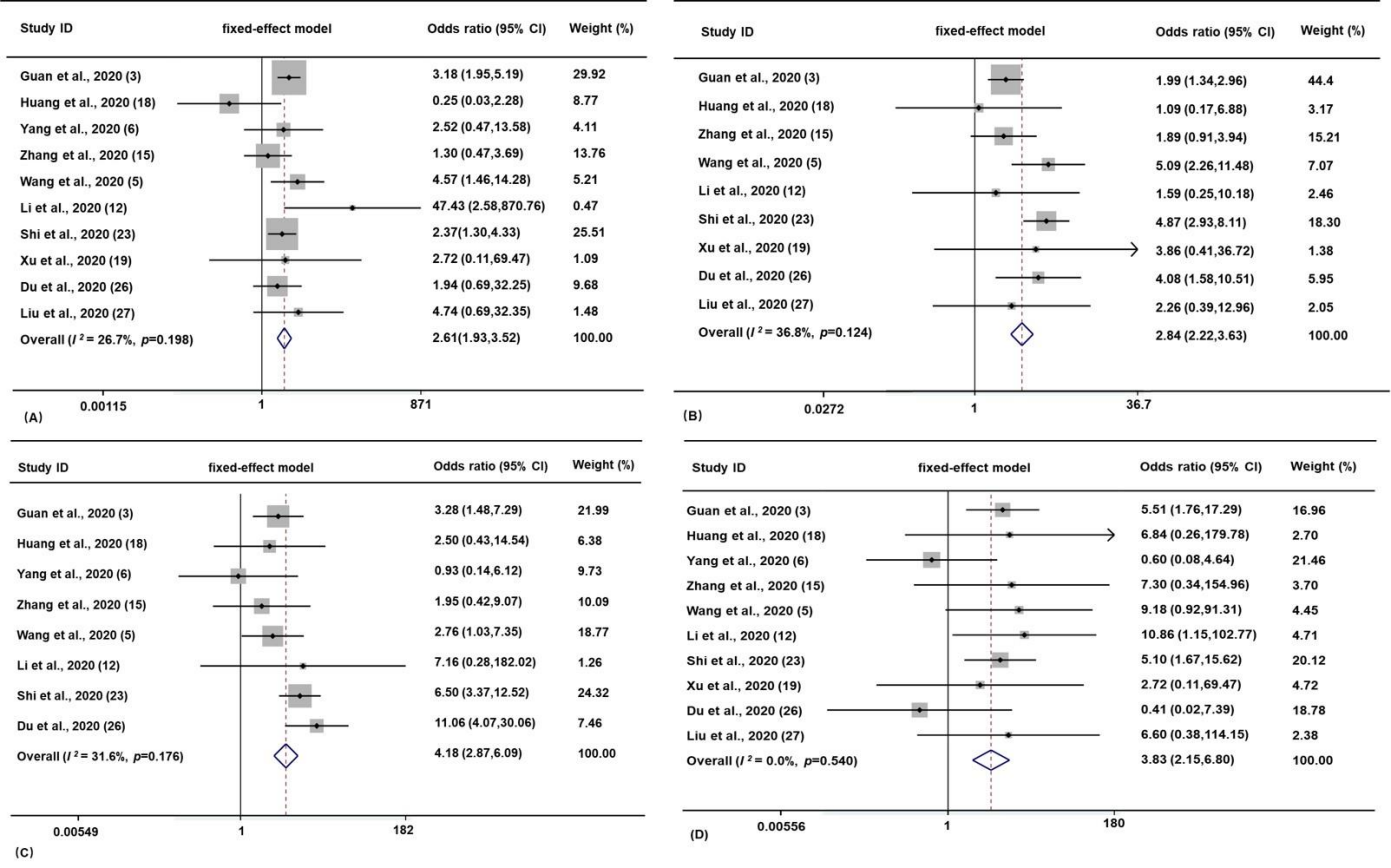
**Correlation between comorbid chronic diseases and severe COVID-19 in SARS-CoV-2 infected patients.** Forest plot showing the effects of comorbid diabetes (**A**), hypertension (**B**), CAD/CVD (**C**), and chronic pulmonary disease (**D**) on the risk of severe COVID-19 in SARS-CoV-2-infected patients. In this figures, the horizontal lines indicate the lower and upper limits of the 95% CI, and the size of the squares reflects the relative weight of each study in the meta-analysis. Weights were calculated from fixed-effects model analyses. Heterogeneity analysis was carried out using Q test and among-studies variation (*I^2^* index).

Next, we found that both preexisting diabetes and hypertension are highly correlated with increased risk of disease severity (OR 2.61, 95% CI 1.93 to 3.52, *I*^2^=26.7%; and OR 2.84, 95% CI 2.22 to 3.63, *I*^2^=36.8%, respectively); and that preexisting CAD/CVD and chronic pulmonary disease are also correlated with disease severity (OR 4.18, 95% CI 2.87 to 6.09, *I*^2^=31.6%; and OR 3.83, 95% CI 2.15 to 6.80, *I*^2^=0.0%, respectively) (Fig. 3, inserts A-D).

### Publication bias

Begg’s test (all Pr >0.05) and Egger’s regression test (all P>0.05) suggest no significant publication bias (Supplementary Fig. 2, inserts A-H).

## DISCUSSION

Recent studies had confirmed that SARS-CoV-2 infection can bring human-to- human transmission and lead to a serious of respiratory, enteric, hepatic, and neurologic damage [32]. SARS-CoV-2 is one type of coronaviruses that belongs to the β-coronavirus cluster. It causes the third kind of zoonotic coronavirus disease after SARS and Middle East respiratory syndrome (MERS) [32]. Both MERS-CoV and SARS-CoV have much higher case fatality rates (40% and 10%, respectively). Though the current SARS-CoV-2 shares 79% of its genome with SARS-CoV, it appears to be much more transmissible [32-33].

The COVID-19 caused a pandemic all over the world. The sex ratio (male to female) was 1.62 observed in the 10948 cases examined in our study. It shows that male was more susceptible to COVID-19. And both sexes exhibited clinical presentations similar in symptomatology. These generally include fever, cough, fatigue or myalgia, breathing difficulty, and diarrhea (Table 1). According to the results of meta-analysis, the top four symptoms of patients with COVID-19 were fever, cough, fatigue or myalgia, and shortness of breath or dyspnea. Diarrhea only occurred among a small number of patients (7.0%, 95% CI 5.0% to 9.0%). However, a recent study reported that diarrhoea was observed in 21% patients with COVID-19, and patients with diarrhoea were older and more likely to have comorbid chronic diseases than patients without diarrhoea [34]. It showed that diarrhoea may be a risk factor for disease severity. Meta-analysis also suggests that people aged more than 50 years occurred 59.0 % (95% CI 49.0% to 68.0 %) during patients with COVID-19 (Table 1). Another meta-analysis showed that elderly people had the highest risk of death during both seasonal and pandemic influenza seasons; in contrast, children and young people aged less than 18 years had a significantly reduced risk of death compared with non-elderly adults during pandemics [35].

Meta-analysis of the data extracted from the included studies (Fig. 2) suggested that diabetes and CAD/CVD were prevalent in 10.0% and 8.0% of the patients, respectively. The proportion of hypertension (20.0%) was much higher than that of chronic pulmonary disease (3.0%) in COVID-19 patients. In April 2016, a study showed that about one-third (32.5%) of Chinese adults had hypertension [35]. The incidence of diabetes, coronary artery disease, and chronic pulmonary disease in China was 10.4%, 1.02%, and 8.0% according to the latest epidemiological data. In conclusion, because hypertension has a much higher prevalence in the population, the incidence of hypertension in patients with COVID-19 was also significantly higher than other comorbid chronic diseases. However, the possible another reason for the low incidence of chronic pulmonary disease in COVID-19 patients is that patients with chronic pulmonary disease may pay more attention to themselves or that virus deposition in the lung could be inhibited by the long-term use of inhaled hormones during patients with asthma. However, another study [36] noted that, patients with chronic pulmonary disease were more likely to need for ICU treatment and ventilator support once they infected pandemic influenza. Our meta-analysis also found that chronic pulmonary disease was a risk factor for increased disease severity in SARS-CoV-2 infected patients. This suggests that the underlying lesions of the lungs in chronic pulmonary disease patients make them more likely to develop into severe cases of influenza or COVID-19. Our finding is consistent with the conclusion of a meta-analysis [37] published a few days ago which included six literatures and found that chronic obstructive pulmonary disease (COPD) was associated with increased disease severity in COVID-19 patients. The sample size included in our present study was larger and the results may be more convincing. Alaa Badawi et al. [38] reported that diabetes and hypertension were equally prevalent in approximately 50%, and CAD/CVD was present in 30% of the severe MERS patients. We also found that the prevalence of diabetes in two studies [24, 29] (cases were from America) was much higher than other 22 studies [3-6, 8, 12-23, 25-28, 30] (cases were from China, Italy, and France) included in our final meta-analysis. However, these could be a reflection of the prevalence of chronic diseases in different regions and races.

In our analysis, we also found that preexisting chronic diseases were strongly correlated with the increased disease severity and increased admittance to ICU. To our surprise, our analysis revealed that preexisting chronic diseases were not significantly correlated with COVID-19 mortality. The possible reason may be the limited literatures. Though there were 24 articles included in our final analysis, only 3 reported the death numbers in COVID-19 patients based on whether there were comorbid chronic diseases. Among the three studies, two studies revealed that compared to COVID-19 patients with no preexisting chronic diseases, COVID-19 patients who present with chronic diseases have a higher rate for mortality; another one study revealed that there was no significant difference in mortality between the two groups. Next, we need more high-quality literatures to confirm the conclusion. The significance of this result is that SARS-CoV-2 infected patients who have preexisting chronic diseases should be hospitalized earlier and get more medical intervention such as ICU admission and mechanical ventilation therapy.

Another study [35] noted that severe pandemic influenza occurred significantly more often in those who were obese (OR 2.74, 95% CI 1.56 to 4.80) and in those who had cardiovascular disease (OR 2.92, 95% CI 1.76 to 4.86), hypertension (OR 1.49, 95% CI 1.10 to 2.01) and neuromuscular disease (OR 2.68, 95% CI 1.91 to 3.75). The comorbid chronic diseases influencing the COVID-19 outcomes may have similar effects in severe MERS and other respiratory illnesses such as influenza H1N1 [39-40]. Common chronic conditions during older people such as diabetes, hypertension, and CAD/CVD, together with their predisposing conditions, may be linked etiologically to the pathogenesis of COVID-19. Chronic diseases share several common features with infectious disorders and their complications, such as endothelial dysfunction, the pro-inflammatory state, and alterations in the innate immune response [41-43]. For example, diabetes and hyperglycemia increase systemic oxidative stress and vascular inflammation. Excessive vascular oxidative stress and vascular inflammation are central characteristics of phonotypical endothelial dysfunction. Chronic diseases associated vascular endothelial dysfunction relates to local vascular inflammation as well as to systemic inflammation. Research also delineate novel roles for elements of both innate and adaptive immune responses in regulating endothelial function under hypertensive conditions. Activation of innate immunity’s complement pathway may negatively impact vascular endothelial function in hypertension [44], whereas increased anti-inflammatory interleukin-10 expression from the adaptive immune response blunts the adverse effects on endothelial function of angiotensin II-associated hypertension [45].

Diabetes mellitus is closely related to the occurrence of severe viral pneumonia and death. For example, in 2003, the mortality of SARS-infected patients who had diabetes mellitus was 3.0-3.3 times higher than that of patients without diabetes mellitus [46, 47]. In 2009, the number of being admitted to ICU in H1N1-infected patients who had diabetes was 4.29 times higher than that of patients without diabetes [48]. Diabetes is also a high risk factor for developing severe MERS in 2014 [48]. Due to abnormal immune functions such as decreased CD3^+ ^T cells, imbalance of CD4^+^/CD8^+ ^T cells and decreased natural killer T cell activity, the immune response ability of the body is decreased [49]. Therefore, preexisting diabetes is a predictive factor for virus infection. On the other hand, angiotensin converting enzyme 2 (ACE2), the functional receptor of SARS-CoV, is also expressed in the islets of the pancreas, so the virus may destroy the islets and aggravate diabetes during infection [50]. SARS-CoV-2 virus can also bind to ACE2 on cells [51]. Virus infection may interact with diabetes, causing SARS-CoV-2 infected patients who had diabetes to be more susceptible to developing severe cases and death.

### Strengths and Limitations of Study

The present study has several limitations. First, the included studies showed a wide variance among studies in the proportion of diabetes, hypertension, CAD/CVD, and chronic pulmonary disease, which may have contributed to the observed significant heterogeneity. Additional sources of heterogeneity may relate to the large variation among studies in the sample size (24 to 5700 patients). Second, most of the studies were from China. These factors may cause some limitations on the estimated contribution of comorbid chronic diseases to COVID-19 cases. Further investigations are needed to examine the nature and extent of coexistence between COVID-19 and non-communicable diseases.

Despite these limitations, our study also has notable strengths. First, this analysis was obtained by pooling data from a number of clinical studies which significantly increased the statistical power of the analysis compared to a single study. Second, the included studies originated from different countries and included different ethnic backgrounds, allowing for the generalization of our results. In conclusion, patients with preexisting chronic diseases may have a higher risk for developing severe COVID-19 and should be given close attention.

## Supplementary Materials

The Supplemenantry data can be found online at: www.aginganddisease.org/EN/10.14336/AD.2020.0502.
