# Supplementary material for: Comorbid Chronic Diseases are Strongly Correlated with Disease Severity among COVID-19 Patients: A Systematic Review and Meta-Analysis
Source: Aging Dis. 2020 May 2;11(3):668–78. doi: 10.14336/AD.2020.0502 (PMC7220287; doi:10.14336/AD.2020.0502)
Supplement: Supplementary file 1 — The Supplemenantry data can be found online at: www.aginganddisease.org/EN/10.14336/AD.2020.0502. [file AD-11-3-668-s.pdf]

SUPPLEMENTARY DATA

Comorbid Chronic Diseases are Strongly Correlated with Disease Severity among COVID-19 Patients: A Systematic Review and Meta-Analysis

Hong Liu[1], Shiyan Chen[2], Min Liu[1], Hao Nie[3], Hongyun Lu[4*]

[1]Department of Nutrition, the Third Xiangya Hospital of Central South University, Changsha, China. [2]Department of Endocrinology & Metabolism, the Fifth Affiliated Hospital of Sun Yat-sen University, Zhuhai, China. [3]Department of Geriatrics, the First Affiliated Hospital of Hunan Normal University, Changsha, China. [4]Department of Endocrinology & Metabolism, Zhuhai Hospital Affiliated with Jinan University, Zhuhai People's Hospital, Zhuhai, China.

© 2020. Liu H et al. Published online at http://www.aginganddisease.org/EN/10.14336/AD.2020.0502

SUPPLEMENTARY DATA

Supplementary Table 1. PRISMA-Checklist.

Section/topic	#	Checklist item	Reported on page #
**TITLE**			
Title	1	Identify the report as a systematic review, meta-analysis, or both.	√
**ABSTRACT**			
Structured summary	2	Provide a structured summary including, as applicable: background; objectives; data sources; study eligibility criteria, participants, and interventions; study appraisal and synthesis methods; results; limitations; conclusions and implications of key findings; systematic review registration number.	√
**INTRODUCTION**			
Rationale	3	Describe the rationale for the review in the context of what is already known.	√
Objectives	4	Provide an explicit statement of questions being addressed with reference to participants, interventions, comparisons, outcomes, and study design (PICOS).	√
**METHODS**			
Protocol and registration	5	Indicate if a review protocol exists, if and where it can be accessed (e.g., Web address), and, if available, provide registration information including registration number.	√
Eligibility criteria	6	Specify study characteristics (e.g., PICOS, length of follow-up) and report characteristics (e.g., years considered, language, publication status) used as criteria for eligibility, giving rationale.	√
Information sources	7	Describe all information sources (e.g., databases with dates of coverage, contact with study authors to identify additional studies) in the search and date last searched.	√
Search	8	Present full electronic search strategy for at least one database, including any limits used, such that it could be repeated.	√
Study selection	9	State the process for selecting studies (i.e., screening, eligibility, included in systematic review, and, if applicable, included in the meta-analysis).	√
Data collection process	10	Describe method of data extraction from reports (e.g., piloted forms, independently, in duplicate) and any processes for obtaining and confirming data from investigators.	√
Data items	11	List and define all variables for which data were sought (e.g., PICOS, funding sources) and any assumptions and simplifications made.	√
Risk of bias in individual studies	12	Describe methods used for assessing risk of bias of individual studies (including specification of whether this was done at the study or outcome level), and how this information is to be used in any data synthesis.	√
Summary measures	13	State the principal summary measures (e.g., risk ratio, difference in means).	√
Synthesis of results	14	Describe the methods of handling data and combining results of studies, if done, including measures of consistency (e.g., I^2) for each meta-analysis.	√
Risk of bias across studies	15	Specify any assessment of risk of bias that may affect the cumulative evidence (e.g., publication bias, selective reporting within studies).	√

© 2020. Liu H et al. Published online at http://www.aginganddisease.org/EN/10.14336/AD.2020.0502

Additional analyses	16	Describe methods of additional analyses (e.g., sensitivity or subgroup analyses, meta-regression), if done, indicating which were pre-specified.	√
**RESULTS**			
Study selection	17	Give numbers of studies screened, assessed for eligibility, and included in the review, with reasons for exclusions at each stage, ideally with a flow diagram.	√
Study characteristics	18	For each study, present characteristics for which data were extracted (e.g., study size, PICOS, follow-up period) and provide the citations.	√
Risk of bias within studies	19	Present data on risk of bias of each study and, if available, any outcome level assessment (see item 12).	√
Results of individual studies	20	For all outcomes considered (benefits or harms), present, for each study: (a) simple summary data for each intervention group (b) effect estimates and confidence intervals, ideally with a forest plot.	√
Synthesis of results	21	Present results of each meta-analysis done, including confidence intervals and measures of consistency.	√
Risk of bias across studies	22	Present results of any assessment of risk of bias across studies (see Item 15).	√
Additional analysis	23	Give results of additional analyses, if done (e.g., sensitivity or subgroup analyses, meta-regression [see Item 16]).	√
**DISCUSSION**			
Summary of evidence	24	Summarize the main findings including the strength of evidence for each main outcome; consider their relevance to key groups (e.g., healthcare providers, users, and policy makers).	√
Limitations	25	Discuss limitations at study and outcome level (e.g., risk of bias), and at review-level (e.g., incomplete retrieval of identified research, reporting bias).	√
Conclusions	26	Provide a general interpretation of the results in the context of other evidence, and implications for future research.	√
**FUNDING**			
Funding	27	Describe sources of funding for the systematic review and other support (e.g., supply of data); role of funders for the systematic review.	√

© 2020. Liu H et al. Published online at http://www.aginganddisease.org/EN/10.14336/AD.2020.0502

SUPPLEMENTARY DATA

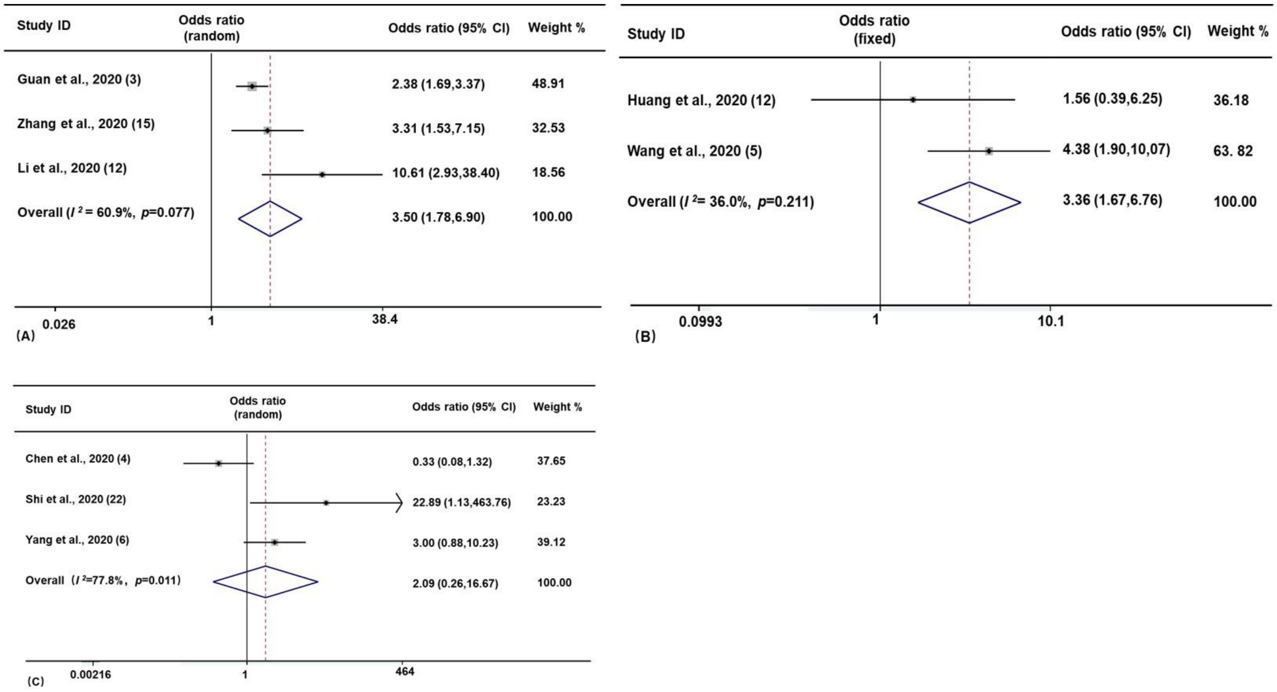

Supplementary Figure 1. Forest plot showing the effects of comorbid diseases on the risk of severe COVID-19 (A), admittance to intensive care unit (ICU) (B), and mortality (C) in SARS-CoV-2-infected patients. In this figures, the horizontal lines indicate the lower and upper limits of the 95% CI, and the size of the squares reflects the relative weight of each study in the meta-analysis. Heterogeneity analysis was carried out using Q test and among-studies variation (I^2 index).

© 2020. Liu H et al. Published online at http://www.aginganddisease.org/EN/10.14336/AD.2020.0502

SUPPLEMENTARY DATA

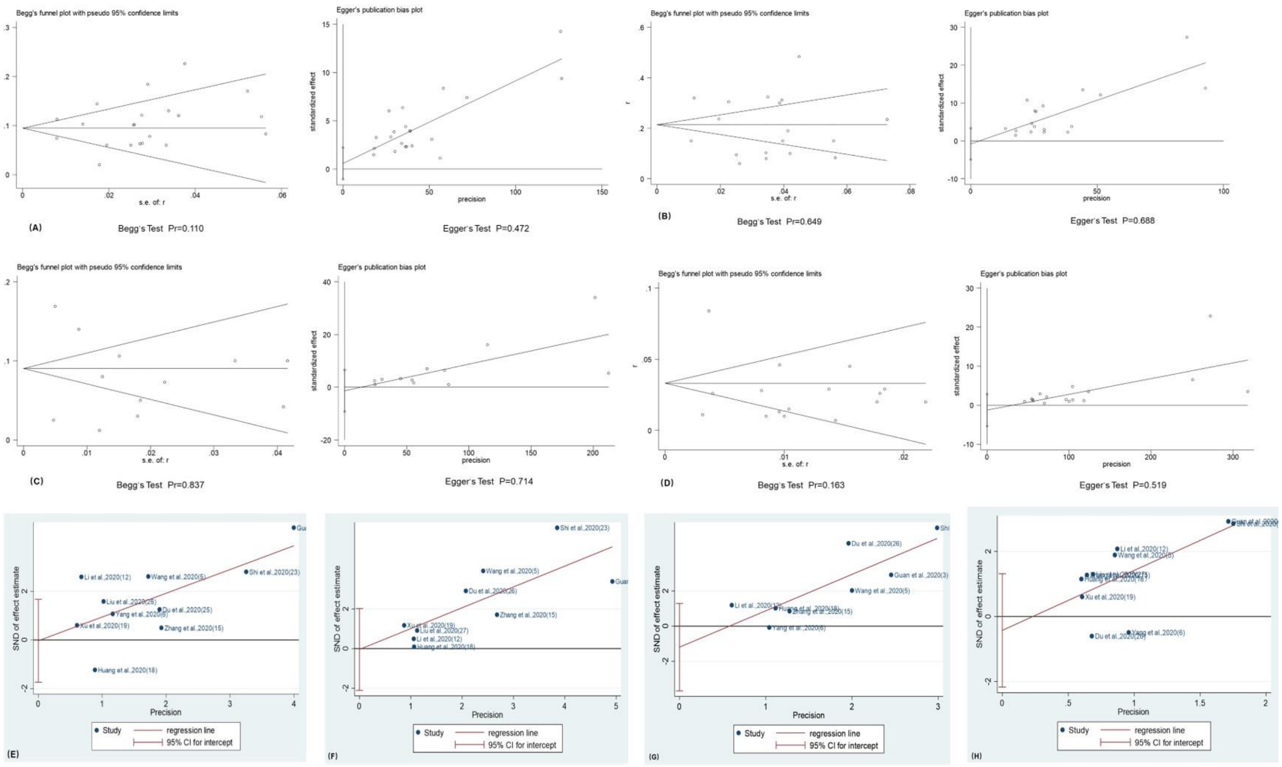

Supplementary Figure 2. The potential for publication bias was addressed by drawing Begg funnel plots and Egger's linear regression test plots; and P<0.05 indicates obvious publication bias. (A)-(D) Prevalence of diabetes, hypertension, coronary artery disease/cardiovascular disease (CAD/CVD), and chronic pulmonary disease during COVID-19 patients, respectively. (A) diabetes, (B) hypertension, (C) CAD/CVD, and (D) chronic pulmonary disease. (E)-(H) The effect of comorbid diabetes, hypertension, CAD/CVD, and chronic pulmonary disease on the risk of severe COVID-19; Egger's test. (E) diabetes, (F) hypertension, (G) CAD/CVD, and (H) chronic pulmonary disease.
